# Colloidal Processing of Mn_3_O_4_-Carbon Nanotube Nanocomposite Electrodes for Supercapacitors

**DOI:** 10.3390/nano12050803

**Published:** 2022-02-26

**Authors:** Wenjuan Yang, Igor Zhitomirsky

**Affiliations:** Department of Materials Science and Engineering, McMaster University, Hamilton, ON L8S 4L7, Canada; yangw48@mcmaster.ca

**Keywords:** manganese, oxide, supercapacitor, nanotube, rhamnolipid, dispersant, nanocomposite, activation, cycling

## Abstract

This investigation addresses the challenges in the development of efficient nanostructured Mn_3_O_4_ cathodes for supercapacitors. A high areal capacitance and the ability to avoid a time-consuming activation procedure for electrodes with high active mass loading of 40 mg cm^−2^ are reported. This facilitates practical applications of Mn_3_O_4_ based electrodes. The highest capacitance of 6.11 F cm^−2^ (153 F g^−1^) is obtained from cyclic voltammetry at a scan rate of 2 mV s^−1^ and 6.07 F cm^−2^ (151.9 F g^−1^) from the chronopotentiometry at a current density of 3 mA cm^−2^ in a potential window of 0.9 V in a neutral Na_2_SO_4_ electrolyte. The new approach is based on the application of rhamnolipids (RL) as a capping agent for the synthesis of Mn_3_O_4_ particles and a co-dispersant for Mn_3_O_4_ and carbon nanotubes, which are used as conductive additives. The size and shape of the Mn_3_O_4_ particles are influenced by RL. The enhanced performance of the electrodes is linked to the chemical structure and properties of RL molecules, which exert influence on Mn_3_O_4_ particle size and shape during synthesis, reduce agglomeration, facilitate RL adsorption on Mn_3_O_4_ and carbon nanotubes, and influence their co-dispersion and mixing at the nanometric scale.

## 1. Introduction

Colloidal methods are widely used for the fabrication of advanced nanomaterials and nanocomposites [1,2,3]. The use of surfactants for colloidal nanofabrication allows efficient control of particle size and prevention of their agglomeration [4,5,6,7]. Of particular interest is the use of surfactants for the fabrication of nanocomposite electrodes for energy storage in supercapacitors. It was found that surfactants facilitate the fabrication of nanoparticles of inorganic charge storage materials with small particle size and prevent their agglomeration [8]. Significant interest has been generated in co-dispersants for efficient mixing of the charge storage materials with conductive additives [8]. The use of such co-dispersants for colloidal fabrication allowed for significant improvement of electrochemical performance of supercapacitors and batteries for practical applications [8,9]. Electrode porosity is an important factor controlling electrochemical performance [10,11,12]. High porosity facilitates good electrolyte access to the active material. Significant attention focused on the development of electrodes with hierarchical porosity [13,14], which allows for superior electrode performance. Advanced techniques were developed for the fabrication of activated carbon, graphene, carbon fiber, MXene, metal oxide and hydroxide electrodes with high porosity [15,16,17,18,19].

This research was motivated by the need in efficient capping agents and dispersants for the fabrication of advanced electrodes for supercapacitors. It has previously been shown that nanocomposites, based on Mn_3_O_4_ are promising materials for cathodes of asymmetric supercapacitors [8]. However, challenges in Mn_3_O_4_ applications are related to the development of efficient electrodes with commercially important high active mass [8]. The specific capacitance decreased with increasing of the active mass [8]. Moreover, the application of Mn_3_O_4_ electrodes with high active mass requires time-consuming activation procedures [20,21,22], which must be avoided for practical applications. It was found that first charge-discharge cycles of Mn_3_O_4_ electrodes showed low capacitance and activation cycling procedure was necessary in order to activate material and achieve high capacitance. Such activation procedures resulted in significant capacitance increase [20,22,23,24]. Several XPS studies revealed oxidation of Mn^2+^ and Mn^3+^ ions on the Mn_3_O_4_ particle surface during cycling and linked this process to the increasing capacitance [20,21,22,25]. The challenges related to Mn_3_O_4_ applications can be addressed using advanced capping agents for the Mn_3_O_4_ synthesis and co-dispersants for Mn_3_O_4_ and conductive additives.

The search for advanced dispersants for colloidal nanotechnology of energy storage materials has generated our interest in rhamnolipids (RL). RL are natural biosurfactants, which offer many benefits since their critical micelle concentration is 10–100 times lower than that of traditional chemical surfactants [26]. RL can solubilize highly hydrophobic organic molecules in aqueous solutions [27]. RL are biocompatible, chemically stable and low cost biosurfactants [26], which have many applications in environmental field, food industry, and biotechnology [28,29,30]. RL are used for prevention of marine oil pollution, removing oil from sand [31] and various applications in agriculture [32,33], laundry products and medicine [26]. RL exhibit valuable antimicrobial and anticancer properties [33,34]. Significant interest has been generated in applications of RL as dispersants for BaTiO_3_ [35], alumina [36,37], zirconia [38], and hematite [39] particles in aqueous suspensions. RL were used as capping agents for synthesis of ZnS [40], NiO [41], and Ag [42] nanoparticles.

The goal of this investigation was the fabrication of Mn_3_O_4_-carbon nanotube composites for cathodes of asymmetric supercapacitors. The use of carbon nanotubes as conductive additives was critically important due to the low electronic conductivity of Mn_3_O_4_ [8,43]. For the first time we report the application of RL as a capping agent for the synthesis of Mn_3_O_4_ nanoparticles. The results presented below indicated that the shape and size of the synthesized Mn_3_O_4_ particles is influenced by RL. Moreover, RL prevent agglomeration of Mn_3_O_4_ particles during synthesis. Another important finding was good co-dispersion of Mn_3_O_4_ and carbon nanotubes by RL, which adsorbed on both materials and facilitated their electrostatic dispersion. It is in this regard that various commercial surfactants are efficient in dispersion of only one type of material, such as inorganic particles or carbon materials [44,45]. The ability of efficient co-dispersion of Mn_3_O_4_ and carbon nanotubes by RL allowed for their efficient mixing and facilitated the fabrication of nanocomposite electrodes with high capacitance. Moreover, the time-consuming activation procedure for the fabrication of Mn_3_O_4_ electrodes can be avoided. The results of this investigation indicated that Mn_3_O_4_-carbon nanotube composites are promising for practical applications for energy storage in cathodes of asymmetric supercapacitors.

## 2. Materials and Methods

RL, ethanol, Mn(NO_3_)_2_·4H_2_O, NaOH, Na_2_SO_4_, poly(vinyl butyral-co-vinyl alcohol-co-vinyl acetate) (PVB, MilliporeSigma, Oakville, ON, Canada), and multiwalled carbon nanotubes (MWCNT, ID 4 nm, OD 13 nm, length 1–2 μm, Bayer, Leverkusen, Germany) were used as starting materials. The as-received MWCNT formed large agglomerates with a typical diameter of 0.5 mm. PVB is advanced co-polymer binder [46,47] designed for colloidal processing of inorganic particles. Polyvinyl alcohol functional groups facilitate PVB adsorption on inorganic particles by formation of hydrogen bonds with hydroxyl groups on the particle surface [46,47]. Butyral segments are directed toward the organic solvent, providing steric stabilization [46,47]. 

Mn_3_O_4_ nanoparticles were prepared by a modified chemical precipitation method [23] and mixed with MWCNT. In method 1, a solution of 330 mg of Mn(NO_3_)_2_·4H_2_O in 20 mL of DI water was prepared and then the pH of the solution was increased to pH = 10 with aqueous NaOH for the Mn_3_O_4_ synthesis. The synthesis was performed without the use of RL. In this method, RL were used as co-dispersants for Mn_3_O_4_ and MWCNT. As-prepared Mn_3_O_4_ was mixed in the aqueous phase with MWCNT and then RL were added. The mass ratio of Mn_3_O_4_:CNT:RL was 4:1:1.

In method 2, RL were used as a capping agent for Mn_3_O_4_ synthesis and a co-dispersant for Mn_3_O_4_ and MWCNT. A solution of 330 mg of Mn(NO_3_)_2_·4H_2_O in DI water was prepared and RL were added as a capping agent for the synthesis of Mn_3_O_4_ nanoparticles to achieve Mn_3_O_4_:RL ratio of 4:2. The pH of the solution was increased to pH = 10 with aqueous NaOH for the Mn_3_O_4_ synthesis and then MWCNT were added to Mn_3_O_4_ in the aqueous phase. The mass ratio of Mn_3_O_4_:CNT:RL was 4:1:2. Additional experiments were performed for mass ratio of Mn_3_O_4_:CNT:RL = 4:1:1 (Figures S1–S4). The mixtures of Mn_3_O_4_ with MWCNT, containing RL and prepared by both methods were ultrasonicated for achieving improved dispersion and mixing, and then washed and dried. In both methods the Mn(NO_3_)_2_ solutions were stirred for 30 min before adding NaOH. The amount of added NaOH was the same in both methods. Obtained powders were used for the fabrication of electrodes using slurries of Mn_3_O_4_ and MWCNT in ethanol, containing PVB as a binder. The PVB binder content was 3% of the total mass of Mn_3_O_4_ and MWCNT. The slurries were used for impregnation of commercial Ni foam (Vale, Toronto, ON, Canada) current collectors. The total mass of impregnated material after drying was 40 mg cm^−2^.

Microstructure investigations were performed using transmission electron microscopy (TEM, Talos 200X microscope, Thermo Scientific, Waltham, MA, USA) and scanning electron microscopy (SEM, JEOL, JSM-7000F microscope, Tokyo, Japan) methods. X-ray diffraction (XRD) analysis (diffractometer Bruker D8, Coventry, UK) was performed using Cu-Kα radiation at the rate of 0.01 degrees per second. Fourier Transform Infrared Spectroscopy (FTIR) studies were performed using a Bruker Vertex 70 spectrometer (Billerica, MA, USA). XPS analysis was performed using Quantera II Scanning XPS instrument (PHI, Chanhassen, MN, USA). Electrochemical studies were performed in aqueous 0.5 M Na_2_SO_4_ electrolyte using PARSTAT 2273 potentiostat (AMETEK, Berwyn, PA, USA) for cyclic voltammetry (CV) and electrochemical impedance spectroscopy (EIS). A BioLogic VMP 300 potentiostat was used for galvanostatic charge-discharge (GCD) investigations (BioLogic, Claix, France). Testing was performed using a 3-electrode electrochemical cell containing a working electrode (impregnated Ni foam), counter-electrode (Pt mesh), and a reference electrode (SCE, saturated calomel electrode). The capacitive properties of electrode material were presented in gravimetric (C_m_, F g^−1^) and areal (C_S_, F cm^−2^) capacitance forms. Capacitances C_m_ and C_S_ were calculated from the CV, EIS and GCD data as it was described in reference [8]. The capacitances calculated from the CV and GCD data represented integral capacitances measured in a potential window of 0–0.9 V versus SCE. The capacitances calculated from the EIS data represented differential capacitances measured at an open circuit potential at voltage amplitude of 5 mV. CV testing procedures (TP) involved obtaining CV at scan rates of 2, 5, 10, 20, 50 and 100 mV s^−1^. EIS measurements were performed after each TP. GCD measurements were performed after the last TP.

## 3. Results and Discussion

**Figure 1:**
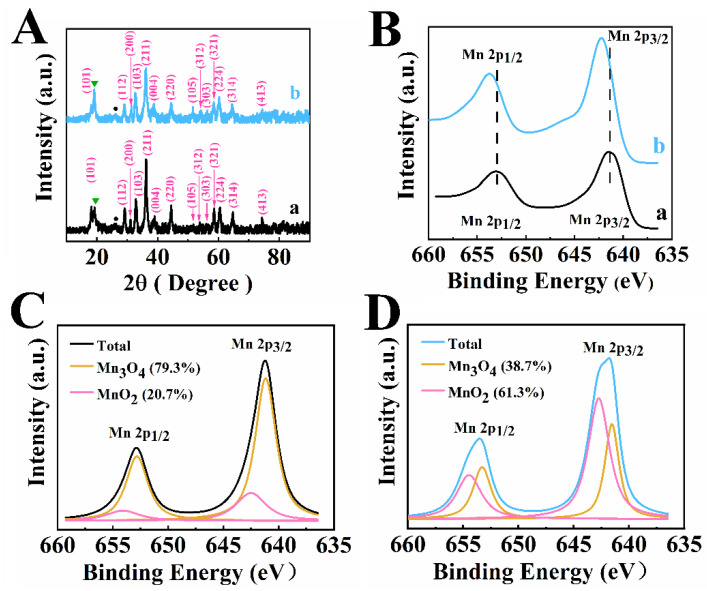
(**A**) X-ray diffraction patterns for Mn_3_O_4_-MWCNT materials prepared by (a) method 1 and (b) method 2, Miller indexes are presented for Mn_3_O_4_ phase, JCPDS file 001-1127, ●—(002) peak of MWCNT, JCPDS file 058-1638,▼—(111) peak of MnO_2_, JCPDS file 083-6090, (**B**–**D**) XPS data for Mn_3_O_4_-MWCNT materials prepared by (**B**(a) and **C**) method 1 and (**B**(b) and **D**) method 2.

Figure 1A shows X-ray diffraction patterns of Mn_3_O_4_-MWCNT composites prepared by methods 1 and 2. The diffraction patterns show major peaks of Mn_3_O_4_, corresponding to the JCPDS file 001-1127 and peaks of MWCNT, corresponding to the JCPDS file 058-1638. The X-ray diffraction pattern of the material prepared by method 1 showed a very small peak of MnO_2_, corresponding to the JCPDS file 083-6090. The relative intensity of this peak was higher for the material prepared by method 2. In this investigation, Mn^2+^ salt was used for the synthesis of manganese oxide. However, Mn^2+^O and Mn^2+^(OH)_2_ are unstable and converted to oxides with higher oxidation state in air [20,21,22,48]. 

The XPS data for the materials prepared by methods 1 and 2 is presented in Figure 1C,D. It should be noted that literature XPS data [49,50,51] for Mn_3_O_4_ showed co-existence of Mn^2+^, Mn^3+^, and Mn^4+^. The peaks corresponding to the 2p_3/2_–2p_1/2_ doublet shifted to higher energies for electrodes, prepared by method 2, compared to the electrodes, prepared by method 1 (Figure 1B). Such shift indicated larger Mn^4+^ content [51,52] in the samples prepared by method 2. A similar shift was observed in Mn_3_O_4_-MnO_2_ hetero-nanorods [52]. Deconvoluted XPS spectra confirmed enlarged MnO_2_ surface content in the samples, prepared by method 2 (Figure 1C,D).

**Figure 2:**
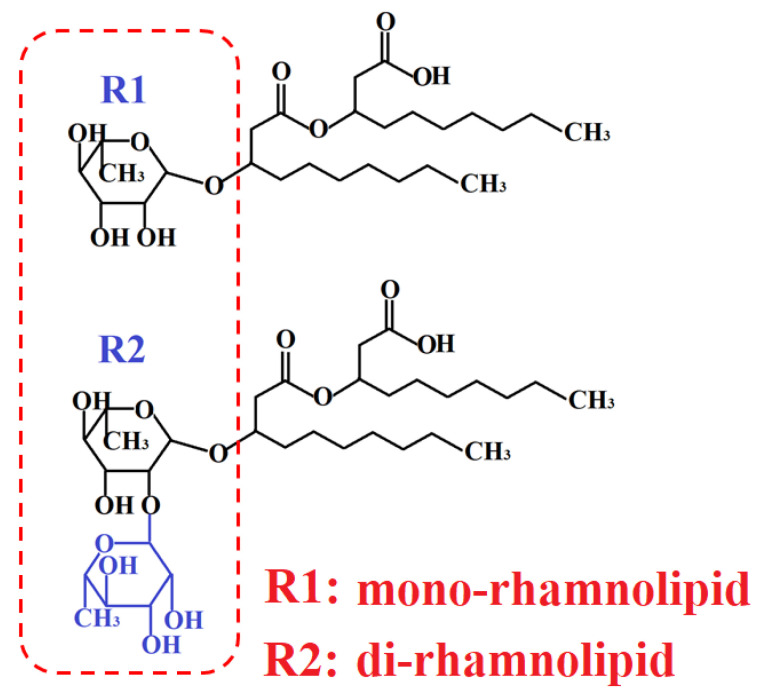
Chemical structure of RL. Dashed line shows rhamnose moieties.

In this investigation RL were used as a capping agent for the synthesis of Mn_3_O_4_ in method 2 and a co-dispersing agent for Mn_3_O_4_ and MWCNT in methods 1 and 2. RL biosurfactants are amphiphilic glycolipids, produced by Pseudomonas aeruginosa [26]. As received RL was a mixture of mono-RL and di-RL. Figure 2 shows chemical structures of RL. The structures contain rhamnose and fatty acid moieties [26]. The amphiphilic structure of RL and electric charge of their carboxylic groups in solutions are important factors, which make RL promising dispersants for electrostatic dispersion of materials. For the investigation of dispersion properties of RL, Mn_3_O_4_ particles were prepared by method 1 without MWCNT, washed, dried, and redispersed in water in the presence of RL with Mn_3_O_4_:RL mass ratio 4:1. MWCNT were dispersed in water in the presence of RL with MWCNT:RL mass ratio of 1:1. Sedimentation tests showed colloidal stability of the obtained suspensions for more than one week. It should be noted that metal oxide nanoparticles often form agglomerates due to their high surface energy. The condensation of surface OH groups also promotes agglomeration. The as-received MWCNT used in this investigation consisted of large agglomerates with a typical size of 0.5 mm [53]. The ability to co-disperse Mn_3_O_4_ and MWCNT using a RL as a co-dispersant is important for their efficient mixing. It is suggested that RL adsorbed on Mn_3_O_4_ and MWCNT and allowed for their electrostatic dispersion. The adsorption of RL on MWCNT resulted from hydrophobic interactions of fatty acid moieties of RL with carbon nanotubes [54]. It is known that RL forms complexes with Mn [55,56]. Therefore, the complexation Mn atoms on the Mn_3_O_4_ particle surface with RL can explain the RL adsorption on Mn_3_O_4_. 

**Figure 3:**
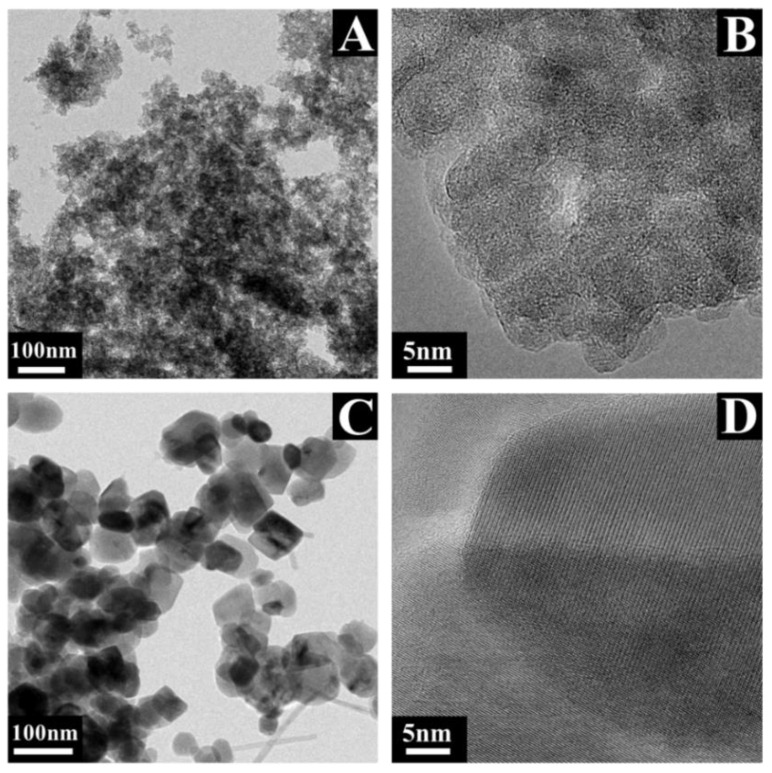
TEM images at different magnifications of Mn_3_O_4_ prepared by method 1 (**A**,**B**) and method 2 (**C**,**D**).

Figure 3 shows TEM images of Mn_3_O_4_ prepared by methods 1 and 2 without MWCNT.

The TEM images of Mn_3_O_4_ prepared by method 1 without a capping agent contained large agglomerates of particles of irregular shape (Figure 3A,B). The morphology of Mn_3_O_4_ particles prepared using RL as a capping agent in method 2 was different (Figure 3C,D). The primary particles were larger and showed crystalline faces. The typical size of the particles was about 50 nm. The particles prepared in the presence of RL as a capping agent showed reduced agglomeration (Figure 3C,D). Therefore, the results of TEM studies showed that the morphology of the synthesized Mn_3_O_4_ was influenced by RL. 

**Figure 4:**
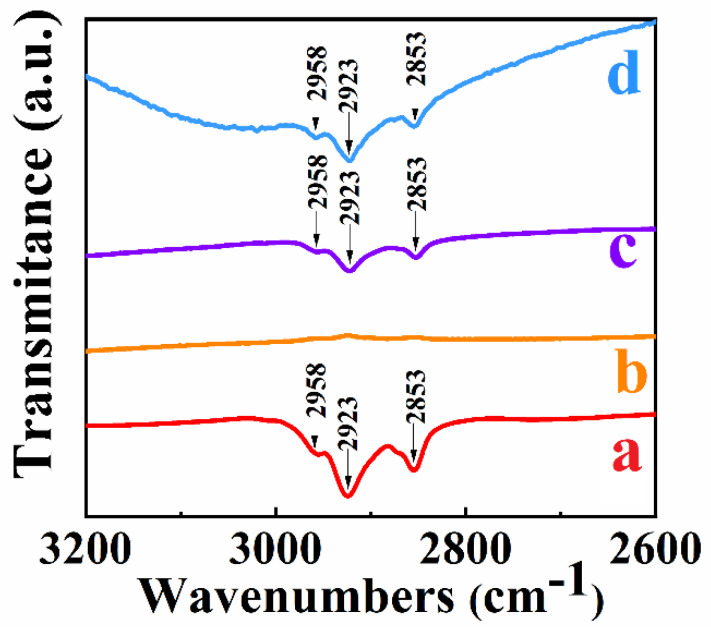
FTIR spectra of (a) as received RL, (b) Mn_3_O_4_ prepared without RL by method 1, (c) Mn_3_O_4_ prepared without RL by method 1 and dispersed using RL, (d) Mn_3_O_4_ prepared using RL as a capping agent by method 2.

FTIR studies were performed to analyze the RL adsorption. The FTIR spectrum of as-received RL (Figure 4a) showed absorptions at 2853, 2923 and 2958 cm^−1^, which can be attributed to the asymmetric and symmetric stretching vibrations of the CH_2_ and CH_3_ groups [57] of RL. Such absorptions were not observed in the spectrum of Mn_3_O_4_ prepared by method 1 without RL (Figure 4b).

For comparison, the Mn_3_O_4_ particles prepared by method 1 were dispersed in the presence of RL. The obtained suspensions were filtered, washed and dried. The FTIR spectrum of obtained powders (Figure 4c) showed absorption peaks, similar to those observed in the spectrum of RL (Figure 4a). Similar absorptions were observed in the spectrum of Mn_3_O_4_ prepared by method 2 (Figure 4d). Therefore, the results of FTIR studies showed that RL absorbed on the Mn_3_O_4_ particles during or after synthesis. 

**Figure 5:**
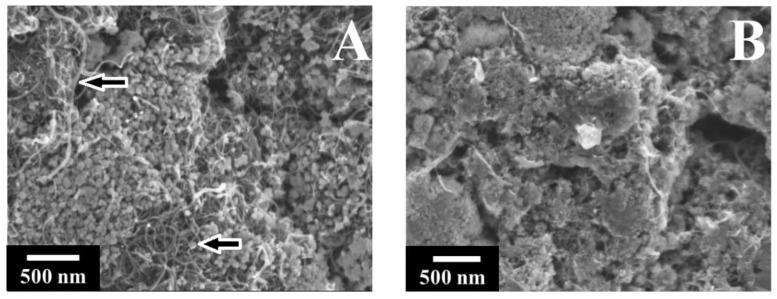
SEM images of electrodes prepared using Mn_3_O_4_-MWCNT composites prepared by (**A**) method 1 and (**B**) method 2. Arrows show areas with enlarged MWCNT content.

**Figure 6:**
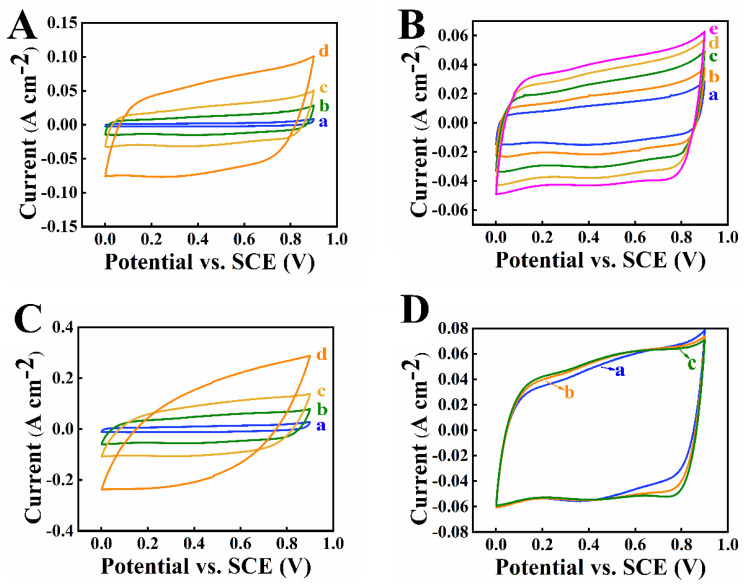
(**A**) CVs at scan rates of (a) 2, (b) 10, (c) 20 and (d) 50 mV s^−1^ for TP1 and (**B**) CVs at a scan rate of 10 mV s^−1^ for (a) TP1, (b) TP2, (c) TP3, (d) TP4 and (e) TP5 for electrode prepared by method 1; (**C**) CVs at scan rates of (a) 2, (b) 10, (c) 20 and (d) 50 mV s^−1^ for TP1 and (**D**) CVs at a scan rate of 10 mV s^−1^ for (a) TP1, (b) TP2, and (c) TP3 for electrode prepared by method 2. Each TP involved testing at scan rates of 2, 5, 10, 20, 50, and 100 mV s^−1^. The CVs for scan rate of 10 mV s^−1^ for each TP were selected and presented in (**B**,**D**).

Figure 5 shows SEM images of composite electrodes, which were fabricated using Mn_3_O_4_-MWCNT composites, prepared by methods 1 and 2. The SEM image of electrodes prepared by method 1 showed that the size of primary Mn_3_O_4_ particles was below 100 nm. However, the Mn_3_O_4_ particles formed agglomerates. This resulted in the areas with larger contents of Mn_3_O_4_ or MWCNT and indicated poor mixing of the components. In contrast, such areas were not observed in the SEM images of the electrodes prepared by method 2, which facilitated improved mixing of Mn_3_O_4_ or MWCNT. CV studies of the electrodes prepared by method 1 showed nearly rectangular CVs for TP 1 (Figure 6A). However, CV areas increased during cycling. Figure 6B presents CVs for TPs 1–5 at a scan rate of 10 mV s^−1^. Significant increase in CV areas indicates increase in capacitance during cycling. This agrees with previous investigations [20,21,22], which showed that time consuming activation is required for Mn_3_O_4_ electrodes with high active mass. Such a time-consuming activation procedure must be avoided for practical applications. Activation of the electrodes prepared by method 1 required 5 TPs and each TP involved testing a scan rates of 2, 5, 10, 20, 50 and 100 mV s^−1^. The electrodes prepared by method 2 showed significantly higher currents for TP 1 (Figure 6C), compared to electrodes prepared by method 1 (Figure 6A). The higher currents indicated higher capacitance. The electrodes prepared by method 2 showed reduced variations in CV areas during cycling. Figure 6D presents CVs at a scan rate of 10 mV s^−1^. Very small variations in CV were observed for TPs 1–3. The CV obtained at TP 3 showed slightly improved rectangular shape, compared to the CV for TP 1.

The areas of CV remained practically without change for TP 4 and TP 5 for electrodes prepared by method 2. CV area for TP 1 for electrode prepared by method 2 (Figure 6(Da)) was larger than the area of CV for TP 5 for the electrode prepared by method 1 (Figure 6(Be)). This indicates higher capacitance of the electrodes prepared by method 2, compared to method 1. Moreover, the need in time consuming activation process can be avoided for electrodes prepared by method 2.

**Figure 7:**
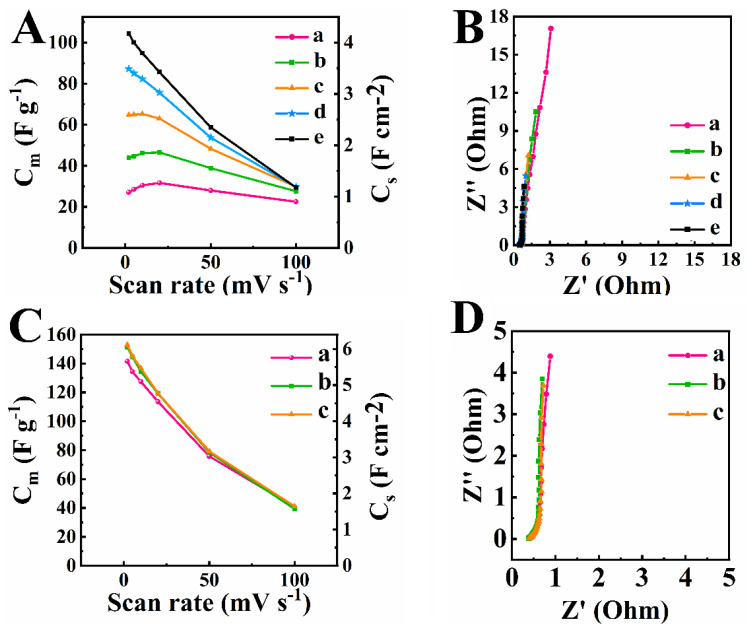
(**A**) Capacitance versus scan rate and (**B**) impedance data presented in a Nyquist plot for (a) TP 1, (b) TP 2, (c) TP 3, (d) TP 4 and (e) TP 5 for electrode prepared by method 1, (**C**) capacitance versus scan rate and (**D**) impedance data presented in a Nyquist plot for (a) TP 1, (b) TP 2, and (c) TP 3 for electrode prepared by method 2.

Figure 7A shows capacitances calculated from the CV data for electrodes prepared by method 1 for TPs 1–5. The capacitance of supercapacitor electrodes usually decreases with increasing scan rate [58] due to the diffusion limitations in pores. However, capacitances for TP 1 and TP 2 showed maxima at a scan rate of 20 mV s^−1^. This can be attributed to electrode activation during initial cycling at low scan rates. Numerous XPS studies showed that the activation process results in oxidation of Mn^2+^ and Mn^3+^ ions on the Mn_3_O_4_ particle surface during cycling in the positive potential range and linked this process to the capacitance increase [20,21,22,25]. The oxidation process was influenced by the duration of the application of a positive potential. Therefore, it is not surprising that the activation process was enhanced at low scan rates.

Capacitance increased and impedance decreased with increasing number of TP (Figure 7A,B). The highest capacitance at a scan rate of 2 mV s^−1^ was found to be 4.14 F cm^−2^ (104.4 F g^−1^) for TP 5 in the method 1. Testing results indicated that activation process is necessary for achieving high capacitance and reducing impedance of electrodes prepared by method 1. As pointed out above, such time-consuming activation process must be avoided for practical applications. 

Electrodes, prepared by method 2, did not show significant variations of capacitance and impedance during cycling. The capacitance obtained for the first cycle of TP 1 at a scan rate of 2 mV s^−1^ was 5.67 F cm^−2^ (141.6 F g^−1^) for electrodes prepared by method 2. It is higher than the capacitance obtained at the same scan rate for TP5 for electrodes prepared by method 1. A capacitance of 6.11 F cm^−2^ (153 F g^−1^) was obtained at a scan rate of 2 mV s^−1^ for TP 3. The real and imaginary parts of impedance for electrode, prepared by method 2 for TP 1 were lower than the corresponding values for TP 5 for electrode, prepared by method 1. This indicated lower resistance and higher capacitance of the electrodes prepared by method 2. The electrodes, prepared by method 2 showed very small variations in capacitance and impedance for TP 4 and TP 5, compared to TP 3.

CV data indicated that significantly higher capacitance was achieved by method 2 and in this method the time-consuming activation procedure can be avoided. This opens an avenue for practical applications of Mn_3_O_4_ based electrodes with high active mass loading. It should be noted that small variations in capacitance were observed in method 2. However, variations in capacitance were also observed for other electrodes, such as MnO_2_ electrodes during initial cycling [59]. Such capacitance increase of the MnO_2_ electrodes was attributed to other factors, such as microstructure changes during initial cycling [59].

**Figure 8:**
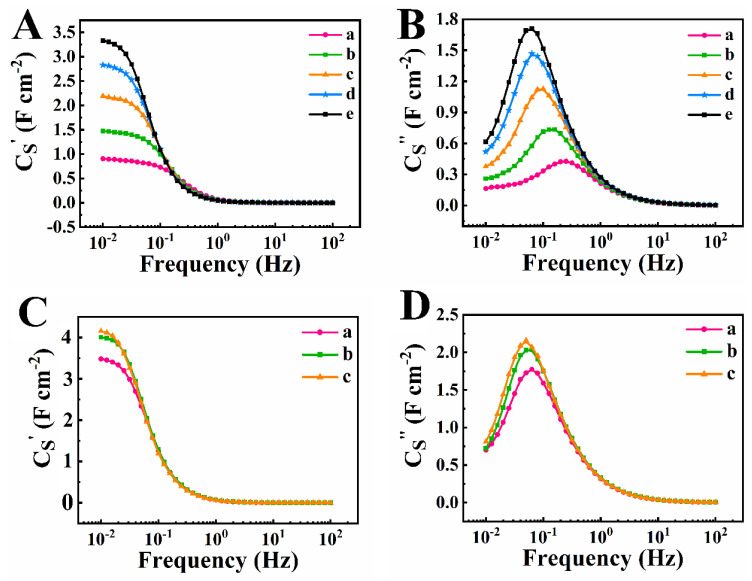
(**A**) real and (**B**) imaginary components of complex capacitance for (a) TP 1, (b) TP 2, (c) TP 3, (d) TP 4 and (e) TP 5 for electrode prepared by method 1, (**C**) real and (**D**) imaginary components of complex capacitance for (a) TP 1, (b) TP 2, (c) TP 3 for electrode prepared by method 2.

Figure 8 shows frequency dependences of real (C_S_′) and imaginary (C_S_″) components of AC capacitance, derived from the impedance data. In contrast to integral capacitance measured by CV method in a potential window of 0.9 V, the components of the differential AC capacitance were measured at voltage amplitude of 5 mV at an open circuit potential.

Figure 8A shows significant increase of low frequency capacitance C_S_′ with increasing TP number for electrodes prepared by method 1. The highest C_S_′ of 3.33 F cm^−2^ was obtained at a frequency of 10 mHz for TP 5. The analysis of frequency dependences of C_S_″ showed significant reduction of the relaxation frequency, corresponding to the C_S_″ maximum with increasing TP number (Figure 8B). The electrode prepared by method 2 showed C_S_′ of 3.48 F cm^−2^ at a frequency of 10 mHz (Figure 8C) for TP 1, which is higher than C_S_′ for electrode prepared by method 1 for TP 5. The C_S_′ increased for TP 2 and showed very small variation for TPs 3–5. The relaxation frequency of the electrodes prepared by method 2 showed very small changes (Figure 8D), especially after TP 2. Therefore, the behavior of the differential capacitance during TPs 1–5 correlated with behavior of the integral capacitance, derived from the CV data.

**Figure 9:**
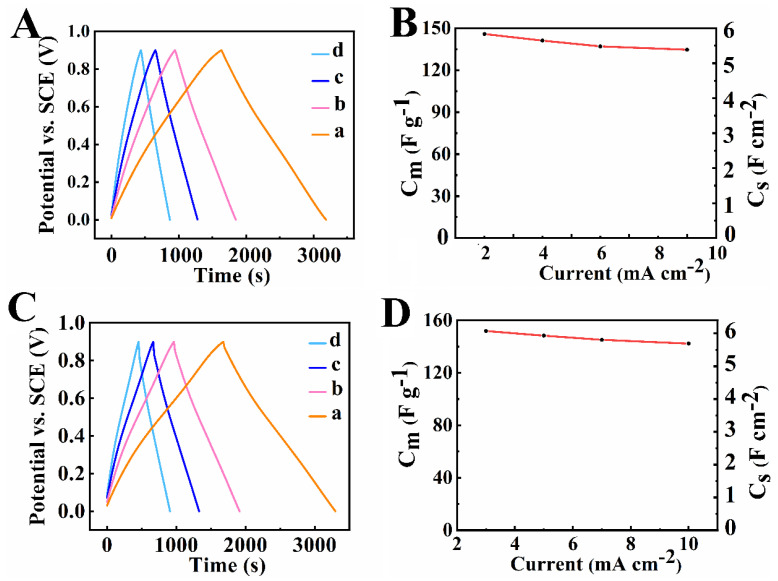
GCD data for electrodes prepared by (**A**,**B**) method 1 and (**C**,**D**) method 2, (**A**,**C**) charge-discharge at current densities of (a) 3, (b) 5, (c) 7, and (d) 10 mA cm^−2^, (**B**,**D**) capacitance versus current density dependences.

The results of the GCD testing of electrodes, prepared by methods 1 and 2, after TP 5 are presented in Figure 9. The GCD curves at different currents showed nearly ideal linear dependences (Figure 9A,C). The electrodes prepared by method 1 and method 2 showed capacitances of 5.83 F cm^−2^ (145.8 F g^−1^) and 6.07 F cm^−2^ (151.9 F g^−1^), respectively, at a current density of 3 mA cm^−2^ (Figure 9B,D). The capacitances showed slight decrease with increasing current density in the range of 3–10 mA cm^−2^.

**Figure 10:**
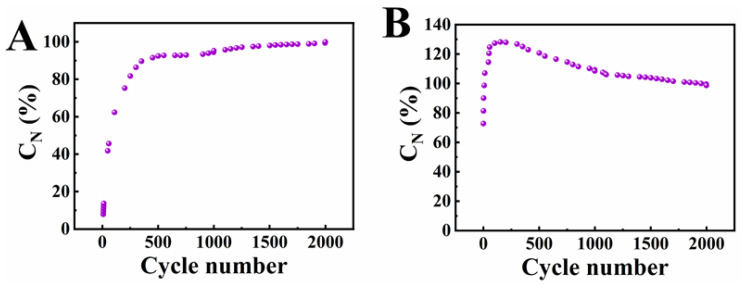
Capacitance (C_N_) normalized by capacitance value for 2000th cycle for electrodes prepared by (**A**) method 1 and (**B**) method 2, obtained from CV data at a scan rate of 50 mV s^−1^.

The influence of cycling on capacitive properties of electrodes prepared by methods 1 and 2 was also studied by analyzing CVs at a scan rate of 50 mV s^−1^. The capacitances for different cycles were normalized by the capacitance obtained at 2000th cycle and presented in Figure 10. The normalized capacitance (C_N_) for electrodes prepared by method 1 was only 8.3% for the cycle 1.

The electrodes prepared by method 1 showed significant increase of C_N_ during first 500 cycles and further continuous capacitance increase at a reduced rate. The electrodes, prepared by method 2 showed C_N_ of 71% for the cycle 1 and C_N_ of 99% for the cycle 7. The C_N_ showed a maximum of 128% for cycle 151 and then decreased. The rate of the C_N_ decrease reduced after 1000 cycles. The CV data provided additional evidence of significantly faster electrode activation in method 2. However, as pointed out above the activation process is also influenced by the scan rate.

Recent comprehensive review [8] of supercapacitor electrodes with high active mass loadings provided a summary of capacitances for Mn_3_O_4_ and MnO_2_ based electrodes. Table 1 shows capacitances of Mn_3_O_4_ electrodes with high active mass loading reported in literature.

It is seen that the areal capacitance achieved in this investigation by method 2 is higher, than that reported in the literature for Mn_3_O_4_ based electrodes of high mass. The method used in this investigation is simple and it is based on the use of a natural co-dispersant. Moreover, the capacitance of Mn_3_O_4_ based electrodes, prepared by method 2 is comparable with capacitance of advanced MnO_2_ based electrodes [8]. The time-consuming activation procedure, which limits the applications of Mn_3_O_4_ based electrodes, can be practically eliminated in method 2. Therefore, Mn_3_O_4_ electrodes represent a promising alternative to the MnO_2_ based electrodes for the development of asymmetric devices for operation in enlarged voltage window in a neutral electrolyte. It should be noted that the application of capping agents, such as RL for the MnO_2_ synthesis presents difficulties due to the use of permanganate precursors, which react with organic additives. It can be expected that Mn_3_O_4_ electrodes with advanced particle morphologies, prepared using capping agents, can outperform MnO_2_ electrodes. Moreover, in contrast to MnO_2_, the spinel type Mn_3_O_4_ forms a large variety of spinel solid solutions. Such solutions can enhance capacitance, reduce resistance, and impart other functional properties, such as ferrimagnetic, catalytic and other properties to the Mn_3_O_4_ based electrodes. 

## 4. Conclusions

For the first time RL were used as a capping agent for the synthesis of Mn_3_O_4_ nanoparticles and as a dispersant for Mn_3_O_4_ and MWCNT. The morphology of the synthesized Mn_3_O_4_ particles and their dispersion were influenced by RL. The chemical structure of RL facilitated their adsorption on materials of different types, such as Mn_3_O_4_ and MWCNT and allowed for their electrostatic dispersion. The ability to co-disperse Mn_3_O_4_ and MWCNT facilitated their efficient mixing at the nanometric scale and allowed for the fabrication of advanced cathode materials for asymmetric supercapacitors. The use of RL as a capping agent resulted in higher capacitance of electrodes prepared by method 2, compared to method 1. The highest capacitance of 6.11 F cm^−2^ (153 F g^−1^) was obtained from CV data at a scan rate of 2 mV s^−1^ and 6.07 F cm^−2^ (151.9 F g^−1^) at a GCD current density of 3 mA cm^−2^ in a potential window of 0.9 V in a neutral Na_2_SO_4_ electrolyte. The problem of time-consuming activation of Mn_3_O_4_ based electrodes can be avoided in the method 2. This makes Mn_3_O_4_ a promising material for practical applications in supercapacitors. Of particular importance for future research is the ability to form spinel solid solutions, based on Mn_3_O_4_. The development of such solid solutions can result in the development of materials with higher capacitance, reduced resistance and multifunctional materials, combining capacitive, ferrimagnetic, catalytic, and other functional properties.

## Figures and Tables

**Table 1:** Literature data on capacitances of Mn_3_O_4_ based electrodes, containing conductive additives, and tested in Na_2_SO_4_ electrolyte.

Active Mass (mg cm^−2^)	Areal Capacitance (F cm^−2^)	Reference
28.4	2.8	[21]
30.4	2.63	[60]
33.0	4.2	[22]
35.0	3.5	[20]
36.0	3.1	[57]
36.0	3.79	[61]
40.1	4.3	[62]
40.0	6.11	this work

## Data Availability

The data presented in this study are available in: “Colloidal Processing of Mn_3_O_4_-Carbon Nanotube Nanocomposite Electrodes for Supercapacitors” and it’s Supplementary information.

## Supplementary Materials

The following is available online at https://www.mdpi.com/article/10.3390/nano12050803/s1. Figure S1: (A) CVs at scan rates of (a) 2, (b) 10, (c) 20, and (d) 50 mV s^−1^ for TP 1 and (B) CVs at a scan rate of 10 mV s^−1^ for (a) TP 1, (b) TP 2, and (c) TP 3 for electrode prepared by method 2; Figure S2: (A) capacitance versus scan rate and (B) impedance data presented in a Nyquist plot for (a) TP 1, (b) TP 2, and (c) TP 3 for electrode prepared by method 2; Figure S3: (A) real and (B) imaginary components of complex capacitance for (a) TP 1, (b) TP 2, (c) TP 3 for electrode prepared by method 2; Figure S4: GCD data for electrodes prepared by method 2, (A) charge-discharge at current densities of (a) 3, (b) 5, (c) 7 and (d) 10 mA cm^−2^, (B) capacitance versus current density dependence.
