# Supplementary material for: Colloidal Processing of Mn3O4-Carbon Nanotube Nanocomposite Electrodes for Supercapacitors
Source: Nanomaterials (Basel). 2022 Feb 26;12(5):803. doi: 10.3390/nano12050803 (PMC8912692; doi:10.3390/nano12050803)
Supplement: Supplementary file 1 [file nanomaterials-12-00803-s001.zip › nanomaterials-1610292-supplementary.pdf]

nanomaterials

Supporting Information

Colloidal Processing of Mn_3O_4-Carbon Nanotube Nanocomposite Electrodes for Supercapacitors

Wenjuan Yang and Igor Zhitomirsky *

Department of Materials Science and Engineering, McMaster University, Hamilton, ON L8S 4L7, Canada; yangw48@mcmaster.ca

* Correspondence: zhitom@mcmaster.ca

Citation: Yang, W.; Zhitomirsky, I. Colloidal processing of Mn_3O_4-Carbon Nanotube Electrodes for Supercapacitors. *Nanomaterials* **2022**, *12*, 803. https://doi.org/10.3390/nano12050803

Publisher's Note: MDPI stays neutral with regard to jurisdictional claims in published maps and institutional affiliations.

Copyright: © 2022 by the authors. Licensee MDPI, Basel, Switzerland. This article is an open access article distributed under the terms and conditions of the Creative Commons Attribution (CC BY) license (https://creativecommons.org/licenses/by/4.0/).

Testing results for Mn_3O_4 electrodes prepared by method 2 with mass ratio Mn_3O_4:MWCNT:RL = 4:1:1

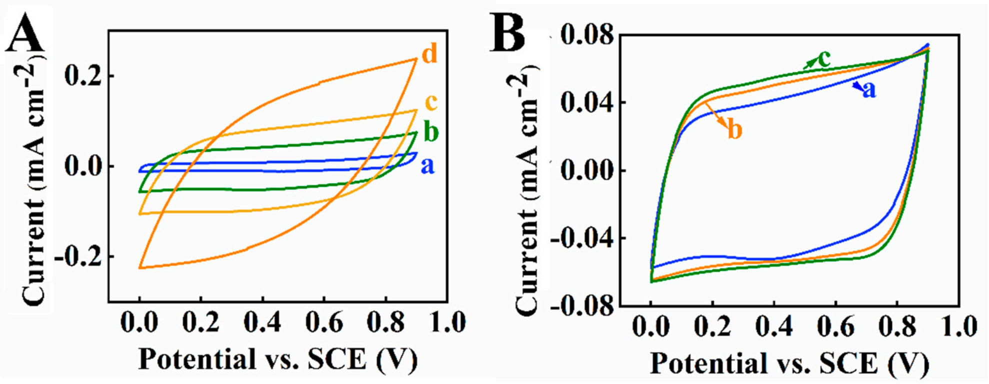

Figure S1. (**A**) CVs at scan rates of (a) 2, (b) 10, (c) 20 and (d) 50 mV s^{-1} for TP 1 and (**B**) CVs at a scan rate of 10 mV s^{-1} for (a) TP 1, (b) TP 2, and (c) TP 3 for electrode prepared by method 2.

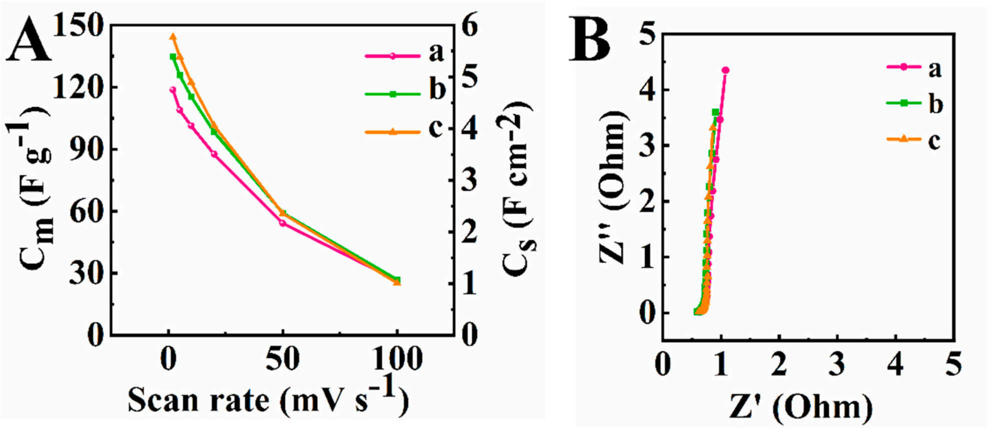

Figure S2. (**A**) capacitance versus scan rate and (**B**) impedance data presented in a Nyquist plot for (a) TP 1, (b) TP 2, and (c) TP 3 for electrode prepared by method 2.

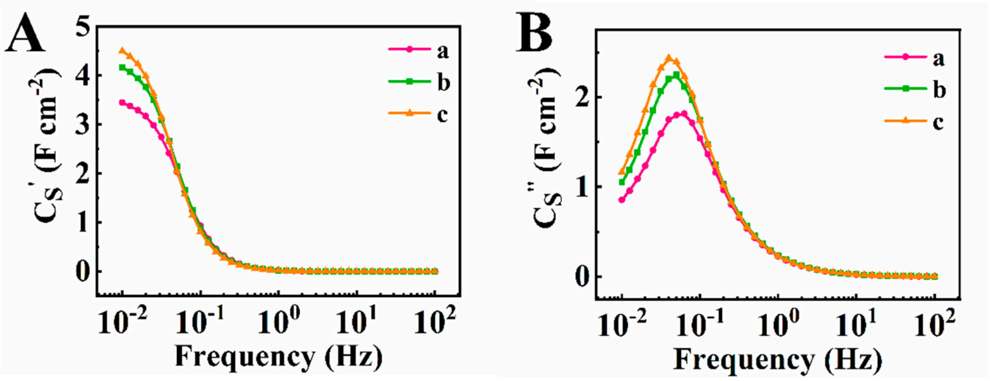

Figure S3. (**A**) real and (**B**) imaginary components of complex capacitance for (a) TP 1, (b) TP 2, (c) TP 3 for electrode prepared by method 2.

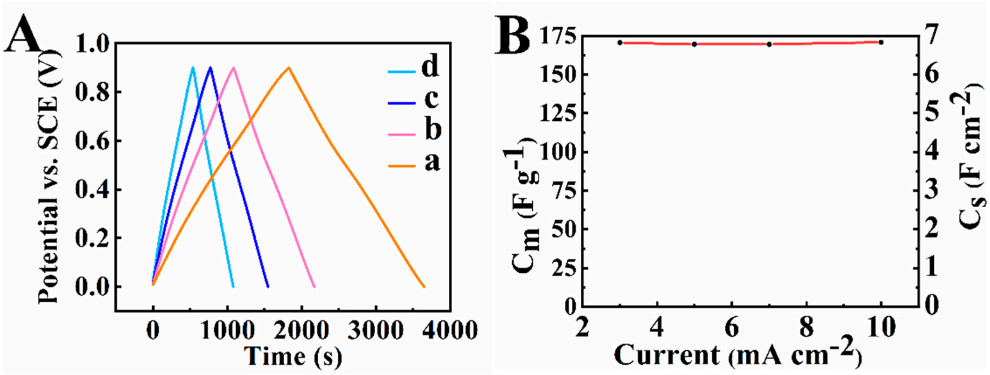

Figure S4. GCD data for electrodes prepared by method 2, (**A**) charge-discharge at current densities of (a) 3, (b) 5, (c) 7 and (d) 10 mA cm^{-2}, (**B**) capacitance versus current density dependence.

Capacitances, calculated from CV data at a scan rate of 2 mV s^{-1} are 4.75 F cm^{-2} (118.8 F g^{-1}), 5.39 F cm^{-2} (134.8 F g^{-1}) and 5.77 F cm^{-2} (144.3 F g^{-1}) for TP 1, TP 2 and TP 3, respectively.

Capacitances, calculated from EIS data at 10 mHz are 4.05 F cm^{-2} (101.3 F g^{-1}), 4.62 F cm^{-2} (115.5 F g^{-1}), 4.90 F cm^{-2} (122.3 F g^{-1}) for TP 1, TP 2 and TP 3, respectively.

Capacitance calculated from the GCD data after TP 5 at a current density of 3 mA cm^{-2} is 6.82 F cm^{-2} (170.5 F g^{-1}).
